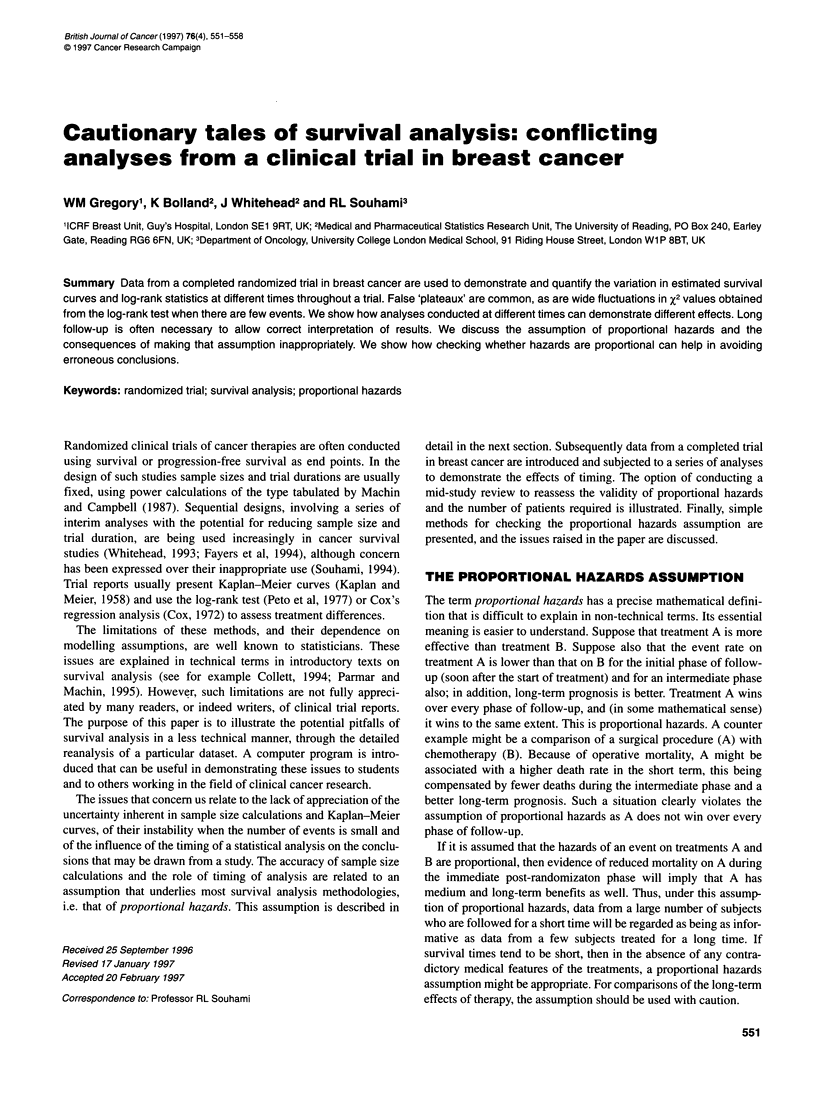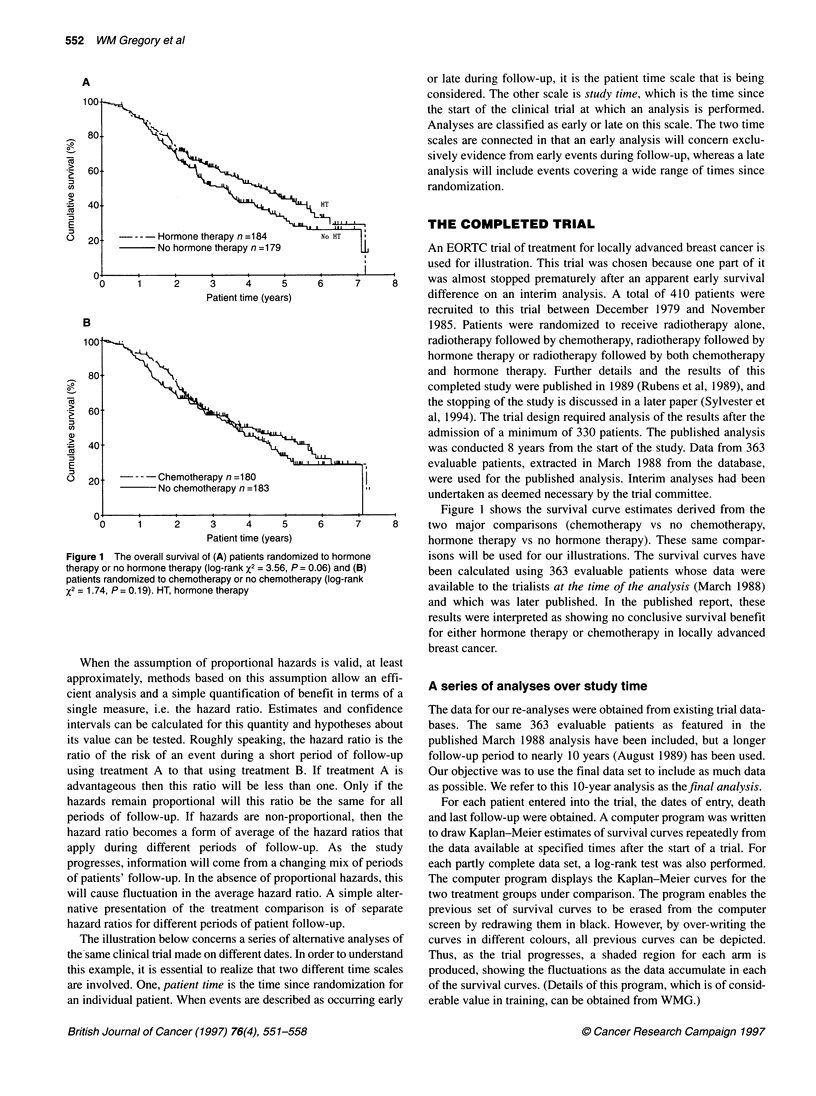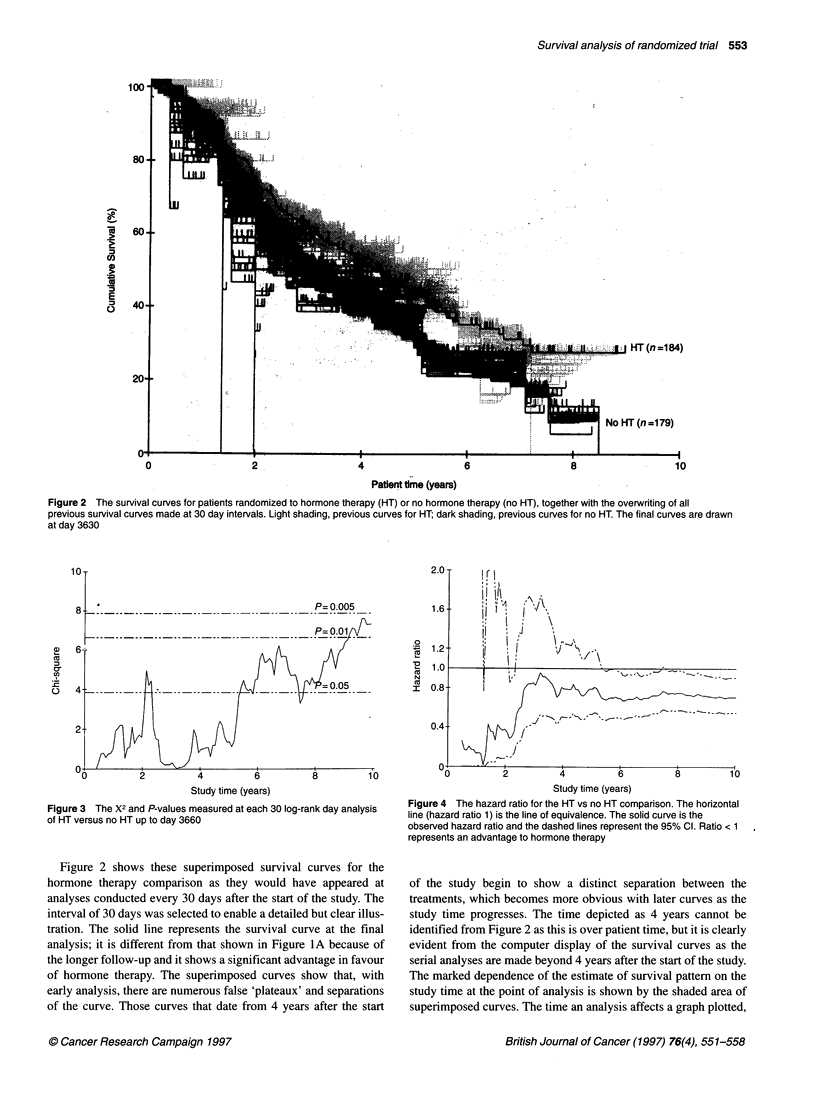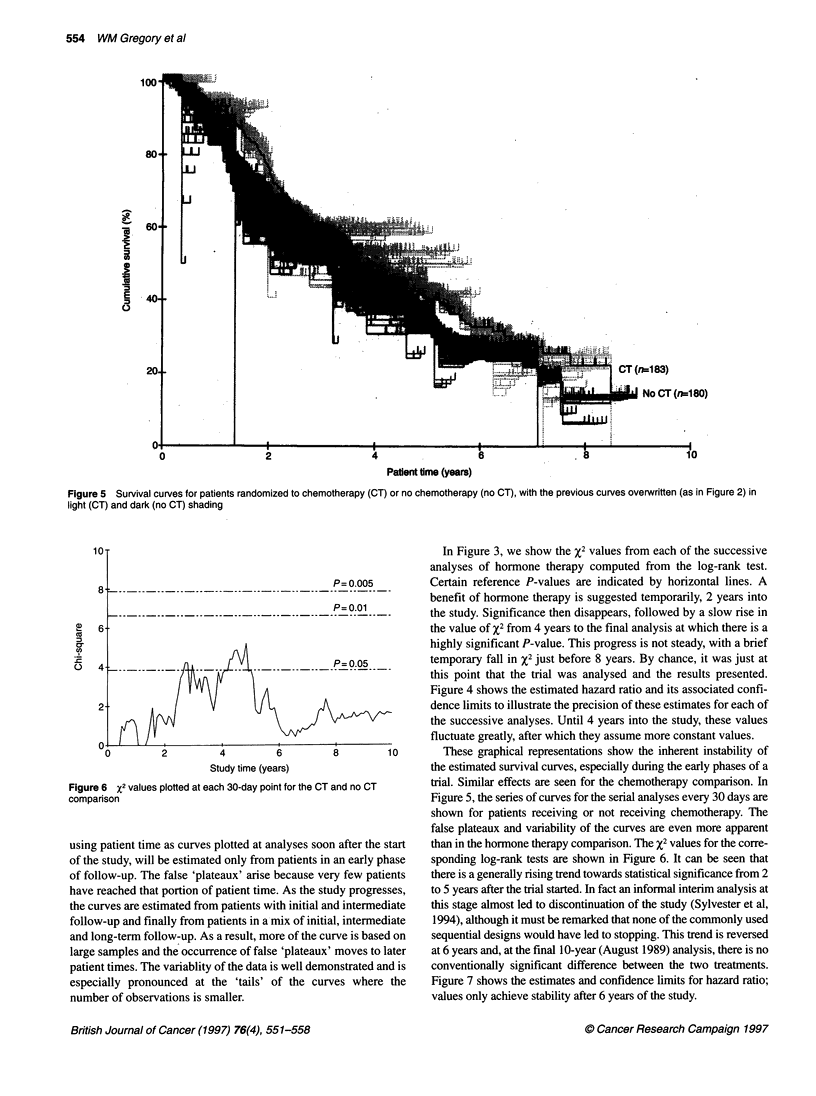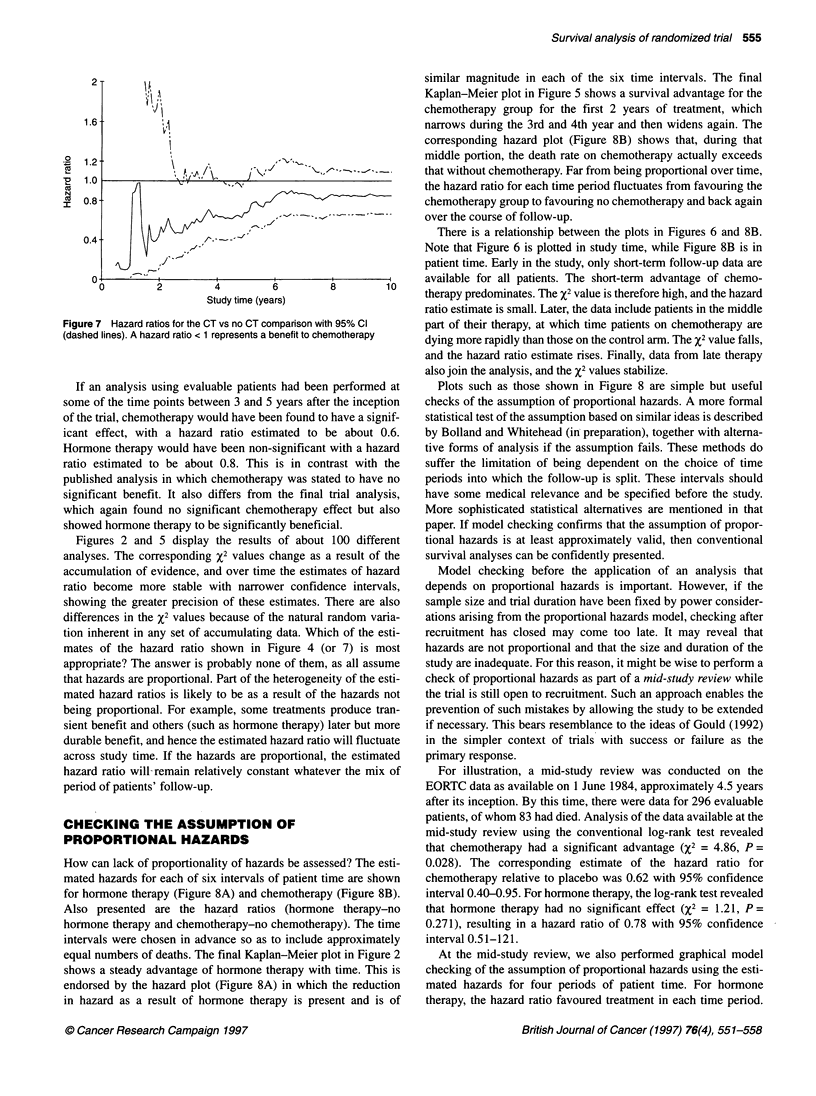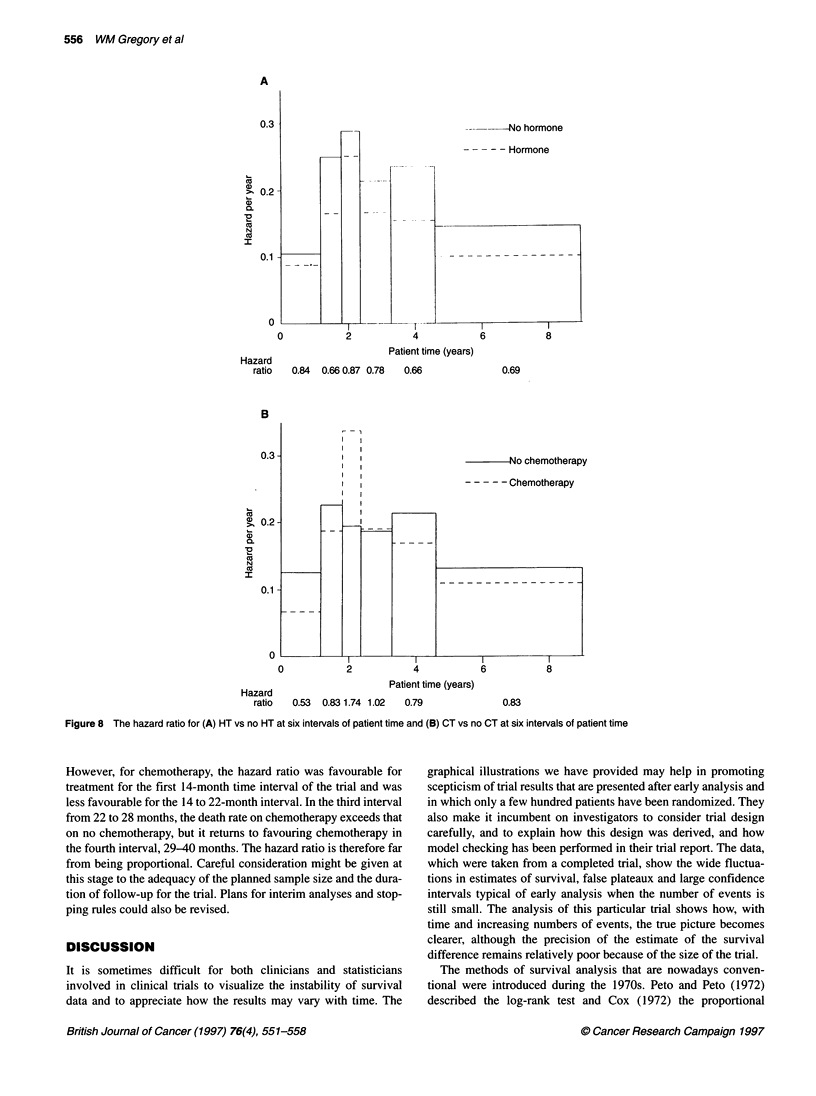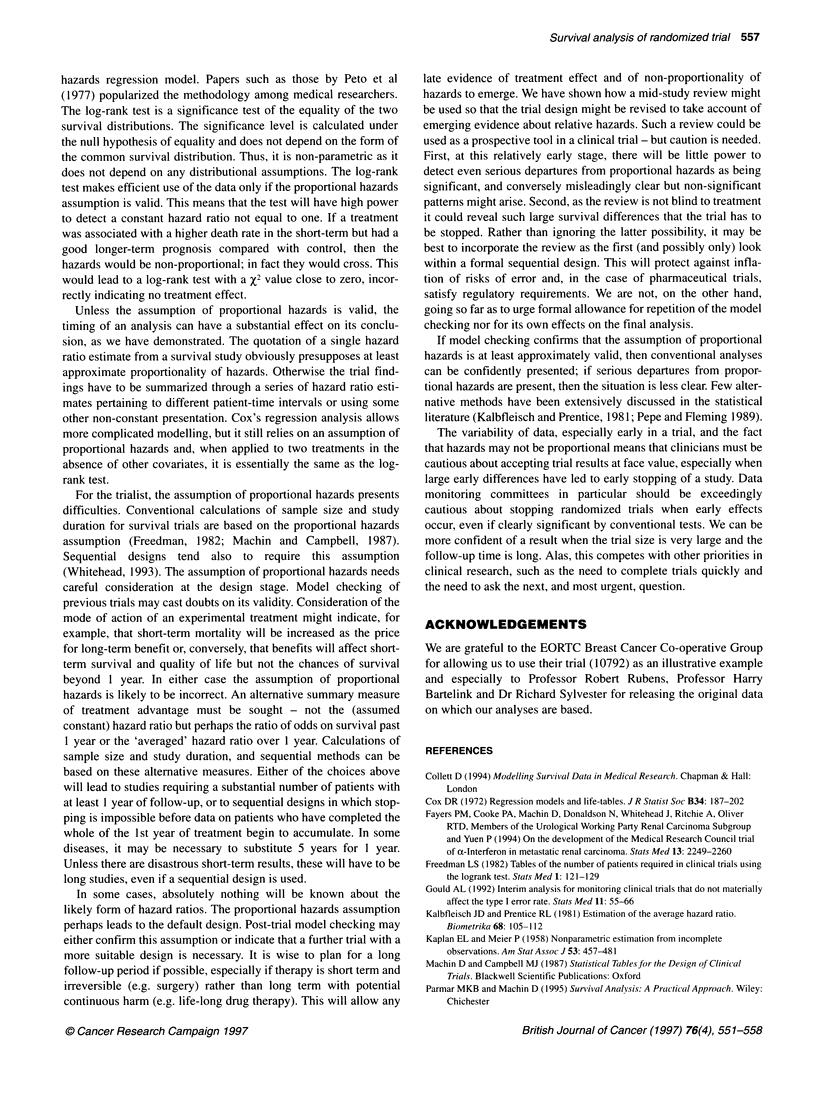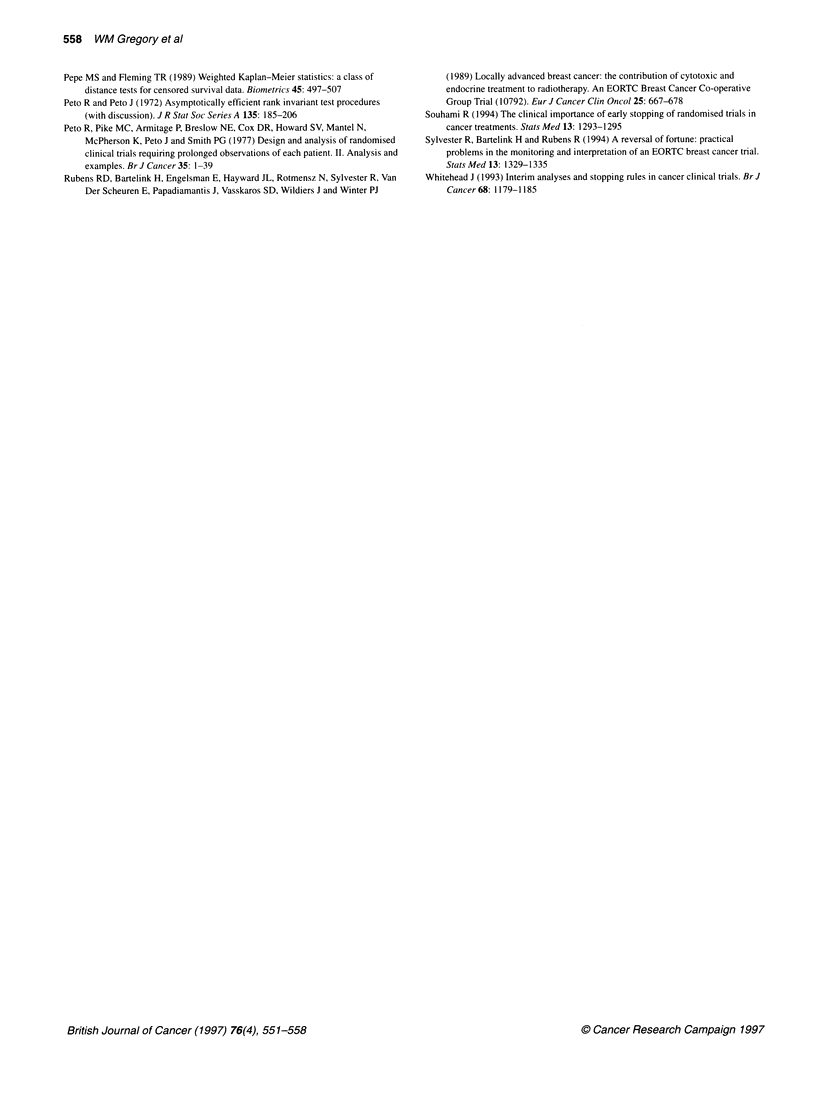# Cautionary tales of survival analysis: conflicting analyses from a clinical trial in breast cancer.

**DOI:** 10.1038/bjc.1997.424

**Published:** 1997

**Authors:** W. M. Gregory, K. Bolland, J. Whitehead, R. L. Souhami

**Affiliations:** ICRF Breast Unit, Guy's Hospital, London, UK.

## Abstract

Data from a completed randomized trial in breast cancer are used to demonstrate and quantify the variation in estimated survival curves and log-rank statistics at different times throughout a trial. False 'plateaux' are common, as are wide fluctuations in chi2 values obtained from the log-rank test when there are few events. We show how analyses conducted at different times can demonstrate different effects. Long follow-up is often necessary to allow correct interpretation of results. We discuss the assumption of proportional hazards and the consequences of making that assumption inappropriately. We show how checking whether hazards are proportional can help in avoiding erroneous conclusions.


British Journal of Cancer (1997) 76(4), 551–558
© 1997 Cancer Research Campaign

### Cautionary tales of survival analysis: conflicting analyses from a clinical trial in breast cancer

**WM Gregory[1], K Bolland[2], J Whitehead[2] and RL Souhami[3]**

[1]ICRF Breast Unit, Guy's Hospital, London SE1 9RT, UK; [2]Medical and Pharmaceutical Statistics Research Unit, The University of Reading, PO Box 240, Earley Gate, Reading RG6 6FN, UK; [3]Department of Oncology, University College London Medical School, 91 Riding House Street, London W1P 8BT, UK

**Summary** Data from a completed randomized trial in breast cancer are used to demonstrate and quantify the variation in estimated survival curves and log-rank statistics at different times throughout a trial. False 'plateaux' are common, as are wide fluctuations in $\chi^2$ values obtained from the log-rank test when there are few events. We show how analyses conducted at different times can demonstrate different effects. Long follow-up is often necessary to allow correct interpretation of results. We discuss the assumption of proportional hazards and the consequences of making that assumption inappropriately. We show how checking whether hazards are proportional can help in avoiding erroneous conclusions.

**Keywords:** randomized trial; survival analysis; proportional hazards

Randomized clinical trials of cancer therapies are often conducted using survival or progression-free survival as end points. In the design of such studies sample sizes and trial durations are usually fixed, using power calculations of the type tabulated by Machin and Campbell (1987). Sequential designs, involving a series of interim analyses with the potential for reducing sample size and trial duration, are being used increasingly in cancer survival studies (Whitehead, 1993; Fayers et al, 1994), although concern has been expressed over their inappropriate use (Souhami, 1994). Trial reports usually present Kaplan–Meier curves (Kaplan and Meier, 1958) and use the log-rank test (Peto et al, 1977) or Cox's regression analysis (Cox, 1972) to assess treatment differences.

The limitations of these methods, and their dependence on modelling assumptions, are well known to statisticians. These issues are explained in technical terms in introductory texts on survival analysis (see for example Collett, 1994; Parmar and Machin, 1995). However, such limitations are not fully appreciated by many readers, or indeed writers, of clinical trial reports. The purpose of this paper is to illustrate the potential pitfalls of survival analysis in a less technical manner, through the detailed reanalysis of a particular dataset. A computer program is introduced that can be useful in demonstrating these issues to students and to others working in the field of clinical cancer research.

The issues that concern us relate to the lack of appreciation of the uncertainty inherent in sample size calculations and Kaplan–Meier curves, of their instability when the number of events is small and of the influence of the timing of a statistical analysis on the conclusions that may be drawn from a study. The accuracy of sample size calculations and the role of timing of analysis are related to an assumption that underlies most survival analysis methodologies, i.e. that of *proportional hazards*. This assumption is described in detail in the next section. Subsequently data from a completed trial in breast cancer are introduced and subjected to a series of analyses to demonstrate the effects of timing. The option of conducting a mid-study review to reassess the validity of proportional hazards and the number of patients required is illustrated. Finally, simple methods for checking the proportional hazards assumption are presented, and the issues raised in the paper are discussed.

*Received 25 September 1996*
*Revised 17 January 1997*
*Accepted 20 February 1997*

*Correspondence to:* Professor RL Souhami

### THE PROPORTIONAL HAZARDS ASSUMPTION

The term *proportional hazards* has a precise mathematical definition that is difficult to explain in non-technical terms. Its essential meaning is easier to understand. Suppose that treatment A is more effective than treatment B. Suppose also that the event rate on treatment A is lower than that on B for the initial phase of follow-up (soon after the start of treatment) and for an intermediate phase also; in addition, long-term prognosis is better. Treatment A wins over every phase of follow-up, and (in some mathematical sense) it wins to the same extent. This is proportional hazards. A counter example might be a comparison of a surgical procedure (A) with chemotherapy (B). Because of operative mortality, A might be associated with a higher death rate in the short term, this being compensated by fewer deaths during the intermediate phase and a better long-term prognosis. Such a situation clearly violates the assumption of proportional hazards as A does not win over every phase of follow-up.

If it is assumed that the hazards of an event on treatments A and B are proportional, then evidence of reduced mortality on A during the immediate post-randomizaton phase will imply that A has medium and long-term benefits as well. Thus, under this assumption of proportional hazards, data from a large number of subjects who are followed for a short time will be regarded as being as informative as data from a few subjects treated for a long time. If survival times tend to be short, then in the absence of any contradictory medical features of the treatments, a proportional hazards assumption might be appropriate. For comparisons of the long-term effects of therapy, the assumption should be used with caution.

**Figure 1** The overall survival of (**A**) patients randomized to hormone therapy or no hormone therapy (log-rank $\chi^2 = 3.56$, $P = 0.06$) and (**B**) patients randomized to chemotherapy or no chemotherapy (log-rank $\chi^2 = 1.74$, $P = 0.19$). HT, hormone therapy

When the assumption of proportional hazards is valid, at least approximately, methods based on this assumption allow an efficient analysis and a simple quantification of benefit in terms of a single measure, i.e. the hazard ratio. Estimates and confidence intervals can be calculated for this quantity and hypotheses about its value can be tested. Roughly speaking, the hazard ratio is the ratio of the risk of an event during a short period of follow-up using treatment A to that using treatment B. If treatment A is advantageous then this ratio will be less than one. Only if the hazards remain proportional will this ratio be the same for all periods of follow-up. If hazards are non-proportional, then the hazard ratio becomes a form of average of the hazard ratios that apply during different periods of follow-up. As the study progresses, information will come from a changing mix of periods of patients' follow-up. In the absence of proportional hazards, this will cause fluctuation in the average hazard ratio. A simple alternative presentation of the treatment comparison is of separate hazard ratios for different periods of patient follow-up.

The illustration below concerns a series of alternative analyses of the same clinical trial made on different dates. In order to understand this example, it is essential to realize that two different time scales are involved. One, *patient time* is the time since randomization for an individual patient. When events are described as occurring early or late during follow-up, it is the patient time scale that is being considered. The other scale is *study time*, which is the time since the start of the clinical trial at which an analysis is performed. Analyses are classified as early or late on this scale. The two time scales are connected in that an early analysis will concern exclusively evidence from early events during follow-up, whereas a late analysis will include events covering a wide range of times since randomization.

### THE COMPLETED TRIAL

An EORTC trial of treatment for locally advanced breast cancer is used for illustration. This trial was chosen because one part of it was almost stopped prematurely after an apparent early survival difference on an interim analysis. A total of 410 patients were recruited to this trial between December 1979 and November 1985. Patients were randomized to receive radiotherapy alone, radiotherapy followed by chemotherapy, radiotherapy followed by hormone therapy or radiotherapy followed by both chemotherapy and hormone therapy. Further details and the results of this completed study were published in 1989 (Rubens et al, 1989), and the stopping of the study is discussed in a later paper (Sylvester et al, 1994). The trial design required analysis of the results after the admission of a minimum of 330 patients. The published analysis was conducted 8 years from the start of the study. Data from 363 evaluable patients, extracted in March 1988 from the database, were used for the published analysis. Interim analyses had been undertaken as deemed necessary by the trial committee.

Figure 1 shows the survival curve estimates derived from the two major comparisons (chemotherapy vs no chemotherapy, hormone therapy vs no hormone therapy). These same comparisons will be used for our illustrations. The survival curves have been calculated using 363 evaluable patients whose data were available to the trialists *at the time of the analysis* (March 1988) and which was later published. In the published report, these results were interpreted as showing no conclusive survival benefit for either hormone therapy or chemotherapy in locally advanced breast cancer.

### A series of analyses over study time

The data for our re-analyses were obtained from existing trial databases. The same 363 evaluable patients as featured in the published March 1988 analysis have been included, but a longer follow-up period to nearly 10 years (August 1989) has been used. Our objective was to use the final data set to include as much data as possible. We refer to this 10-year analysis as the *final analysis*.

For each patient entered into the trial, the dates of entry, death and last follow-up were obtained. A computer program was written to draw Kaplan–Meier estimates of survival curves repeatedly from the data available at specified times after the start of a trial. For each partly complete data set, a log-rank test was also performed. The computer program displays the Kaplan–Meier curves for the two treatment groups under comparison. The program enables the previous set of survival curves to be erased from the computer screen by redrawing them in black. However, by over-writing the curves in different colours, all previous curves can be depicted. Thus, as the trial progresses, a shaded region for each arm is produced, showing the fluctuations as the data accumulate in each of the survival curves. (Details of this program, which is of considerable value in training, can be obtained from WMG.)

 © Cancer Research Campaign 1997

**Figure 2** The survival curves for patients randomized to hormone therapy (HT) or no hormone therapy (no HT), together with the overwriting of all previous survival curves made at 30 day intervals. Light shading, previous curves for HT; dark shading, previous curves for no HT. The final curves are drawn at day 3630

**Figure 3** The $X^2$ and *P*-values measured at each 30 log-rank day analysis of HT versus no HT up to day 3660

**Figure 4** The hazard ratio for the HT vs no HT comparison. The horizontal line (hazard ratio 1) is the line of equivalence. The solid curve is the observed hazard ratio and the dashed lines represent the 95% CI. Ratio < 1 represents an advantage to hormone therapy

Figure 2 shows these superimposed survival curves for the hormone therapy comparison as they would have appeared at analyses conducted every 30 days after the start of the study. The interval of 30 days was selected to enable a detailed but clear illustration. The solid line represents the survival curve at the final analysis; it is different from that shown in Figure 1A because of the longer follow-up and it shows a significant advantage in favour of hormone therapy. The superimposed curves show that, with early analysis, there are numerous false 'plateaux' and separations of the curve. Those curves that date from 4 years after the start of the study begin to show a distinct separation between the treatments, which becomes more obvious with later curves as the study time progresses. The time depicted as 4 years cannot be identified from Figure 2 as this is over patient time, but it is clearly evident from the computer display of the survival curves as the serial analyses are made beyond 4 years after the start of the study. The marked dependence of the estimate of survival pattern on the study time at the point of analysis is shown by the shaded area of superimposed curves. The time an analysis affects a graph plotted,

**Figure 5** Survival curves for patients randomized to chemotherapy (CT) or no chemotherapy (no CT), with the previous curves overwritten (as in Figure 2) in light (CT) and dark (no CT) shading

**Figure 6** $\chi^2$ values plotted at each 30-day point for the CT and no CT comparison using patient time as curves plotted at analyses soon after the start of the study, will be estimated only from patients in an early phase of follow-up. The false 'plateaux' arise because very few patients have reached that portion of patient time. As the study progresses, the curves are estimated from patients with initial and intermediate follow-up and finally from patients in a mix of initial, intermediate and long-term follow-up. As a result, more of the curve is based on large samples and the occurrence of false 'plateaux' moves to later patient times. The variablity of the data is well demonstrated and is especially pronounced at the 'tails' of the curves where the number of observations is smaller.

In Figure 3, we show the $\chi^2$ values from each of the successive analyses of hormone therapy computed from the log-rank test. Certain reference $P$-values are indicated by horizontal lines. A benefit of hormone therapy is suggested temporarily, 2 years into the study. Significance then disappears, followed by a slow rise in the value of $\chi^2$ from 4 years to the final analysis at which there is a highly significant $P$-value. This progress is not steady, with a brief temporary fall in $\chi^2$ just before 8 years. By chance, it was just at this point that the trial was analysed and the results presented. Figure 4 shows the estimated hazard ratio and its associated confidence limits to illustrate the precision of these estimates for each of the successive analyses. Until 4 years into the study, these values fluctuate greatly, after which they assume more constant values.

These graphical representations show the inherent instability of the estimated survival curves, especially during the early phases of a trial. Similar effects are seen for the chemotherapy comparison. In Figure 5, the series of curves for the serial analyses every 30 days are shown for patients receiving or not receiving chemotherapy. The false plateaux and variability of the curves are even more apparent than in the hormone therapy comparison. The $\chi^2$ values for the corresponding log-rank tests are shown in Figure 6. It can be seen that there is a generally rising trend towards statistical significance from 2 to 5 years after the trial started. In fact an informal interim analysis at this stage almost led to discontinuation of the study (Sylvester et al, 1994), although it must be remarked that none of the commonly used sequential designs would have led to stopping. This trend is reversed at 6 years and, at the final 10-year (August 1989) analysis, there is no conventionally significant difference between the two treatments. Figure 7 shows the estimates and confidence limits for hazard ratio; values only achieve stability after 6 years of the study.

© Cancer Research Campaign 1997

**Figure 7** Hazard ratios for the CT vs no CT comparison with 95% CI (dashed lines). A hazard ratio < 1 represents a benefit to chemotherapy

If an analysis using evaluable patients had been performed at some of the time points between 3 and 5 years after the inception of the trial, chemotherapy would have been found to have a significant effect, with a hazard ratio estimated to be about 0.6. Hormone therapy would have been non-significant with a hazard ratio estimated to be about 0.8. This is in contrast with the published analysis in which chemotherapy was stated to have no significant benefit. It also differs from the final trial analysis, which again found no significant chemotherapy effect but also showed hormone therapy to be significantly beneficial.

Figures 2 and 5 display the results of about 100 different analyses. The corresponding $\chi^2$ values change as a result of the accumulation of evidence, and over time the estimates of hazard ratio become more stable with narrower confidence intervals, showing the greater precision of these estimates. There are also differences in the $\chi^2$ values because of the natural random variation inherent in any set of accumulating data. Which of the estimates of the hazard ratio shown in Figure 4 (or 7) is most appropriate? The answer is probably none of them, as all assume that hazards are proportional. Part of the heterogeneity of the estimated hazard ratios is likely to be as a result of the hazards not being proportional. For example, some treatments produce transient benefit and others (such as hormone therapy) later but more durable benefit, and hence the estimated hazard ratio will fluctuate across study time. If the hazards are proportional, the estimated hazard ratio will remain relatively constant whatever the mix of period of patients' follow-up.

### CHECKING THE ASSUMPTION OF PROPORTIONAL HAZARDS

How can lack of proportionality of hazards be assessed? The estimated hazards for each of six intervals of patient time are shown for hormone therapy (Figure 8A) and chemotherapy (Figure 8B). Also presented are the hazard ratios (hormone therapy–no hormone therapy and chemotherapy–no chemotherapy). The time intervals were chosen in advance so as to include approximately equal numbers of deaths. The final Kaplan–Meier plot in Figure 2 shows a steady advantage of hormone therapy with time. This is endorsed by the hazard plot (Figure 8A) in which the reduction in hazard as a result of hormone therapy is present and is of similar magnitude in each of the six time intervals. The final Kaplan–Meier plot in Figure 5 shows a survival advantage for the chemotherapy group for the first 2 years of treatment, which narrows during the 3rd and 4th year and then widens again. The corresponding hazard plot (Figure 8B) shows that, during that middle portion, the death rate on chemotherapy actually exceeds that without chemotherapy. Far from being proportional over time, the hazard ratio for each time period fluctuates from favouring the chemotherapy group to favouring no chemotherapy and back again over the course of follow-up.

There is a relationship between the plots in Figures 6 and 8B. Note that Figure 6 is plotted in study time, while Figure 8B is in patient time. Early in the study, only short-term follow-up data are available for all patients. The short-term advantage of chemotherapy predominates. The $\chi^2$ value is therefore high, and the hazard ratio estimate is small. Later, the data include patients in the middle part of their therapy, at which time patients on chemotherapy are dying more rapidly than those on the control arm. The $\chi^2$ value falls, and the hazard ratio estimate rises. Finally, data from late therapy also join the analysis, and the $\chi^2$ values stabilize.

Plots such as those shown in Figure 8 are simple but useful checks of the assumption of proportional hazards. A more formal statistical test of the assumption based on similar ideas is described by Bolland and Whitehead (in preparation), together with alternative forms of analysis if the assumption fails. These methods do suffer the limitation of being dependent on the choice of time periods into which the follow-up is split. These intervals should have some medical relevance and be specified before the study. More sophisticated statistical alternatives are mentioned in that paper. If model checking confirms that the assumption of proportional hazards is at least approximately valid, then conventional survival analyses can be confidently presented.

Model checking before the application of an analysis that depends on proportional hazards is important. However, if the sample size and trial duration have been fixed by power considerations arising from the proportional hazards model, checking after recruitment has closed may come too late. It may reveal that hazards are not proportional and that the size and duration of the study are inadequate. For this reason, it might be wise to perform a check of proportional hazards as part of a *mid-study review* while the trial is still open to recruitment. Such an approach enables the prevention of such mistakes by allowing the study to be extended if necessary. This bears resemblance to the ideas of Gould (1992) in the simpler context of trials with success or failure as the primary response.

For illustration, a mid-study review was conducted on the EORTC data as available on 1 June 1984, approximately 4.5 years after its inception. By this time, there were data for 296 evaluable patients, of whom 83 had died. Analysis of the data available at the mid-study review using the conventional log-rank test revealed that chemotherapy had a significant advantage ($\chi^2$ = 4.86, $P$ = 0.028). The corresponding estimate of the hazard ratio for chemotherapy relative to placebo was 0.62 with 95% confidence interval 0.40–0.95. For hormone therapy, the log-rank test revealed that hormone therapy had no significant effect ($\chi^2$ = 1.21, $P$ = 0.271), resulting in a hazard ratio of 0.78 with 95% confidence interval 0.51–121.

At the mid-study review, we also performed graphical model checking of the assumption of proportional hazards using the estimated hazards for four periods of patient time. For hormone therapy, the hazard ratio favoured treatment in each time period.

© Cancer Research Campaign 1997 

**Figure 8** The hazard ratio for (**A**) HT vs no HT at six intervals of patient time and (**B**) CT vs no CT at six intervals of patient time

However, for chemotherapy, the hazard ratio was favourable for treatment for the first 14-month time interval of the trial and was less favourable for the 14 to 22-month interval. In the third interval from 22 to 28 months, the death rate on chemotherapy exceeds that on no chemotherapy, but it returns to favouring chemotherapy in the fourth interval, 29–40 months. The hazard ratio is therefore far from being proportional. Careful consideration might be given at this stage to the adequacy of the planned sample size and the duration of follow-up for the trial. Plans for interim analyses and stopping rules could also be revised.

### DISCUSSION

It is sometimes difficult for both clinicians and statisticians involved in clinical trials to visualize the instability of survival data and to appreciate how the results may vary with time. The graphical illustrations we have provided may help in promoting scepticism of trial results that are presented after early analysis and in which only a few hundred patients have been randomized. They also make it incumbent on investigators to consider trial design carefully, and to explain how this design was derived, and how model checking has been performed in their trial report. The data, which were taken from a completed trial, show the wide fluctuations in estimates of survival, false plateaux and large confidence intervals typical of early analysis when the number of events is still small. The analysis of this particular trial shows how, with time and increasing numbers of events, the true picture becomes clearer, although the precision of the estimate of the survival difference remains relatively poor because of the size of the trial.

The methods of survival analysis that are nowadays conventional were introduced during the 1970s. Peto and Peto (1972) described the log-rank test and Cox (1972) the proportional

© Cancer Research Campaign 1997

hazards regression model. Papers such as those by Peto et al (1977) popularized the methodology among medical researchers. The log-rank test is a significance test of the equality of the two survival distributions. The significance level is calculated under the null hypothesis of equality and does not depend on the form of the common survival distribution. Thus, it is non-parametric as it does not depend on any distributional assumptions. The log-rank test makes efficient use of the data only if the proportional hazards assumption is valid. This means that the test will have high power to detect a constant hazard ratio not equal to one. If a treatment was associated with a higher death rate in the short-term but had a good longer-term prognosis compared with control, then the hazards would be non-proportional; in fact they would cross. This would lead to a log-rank test with a $\chi^2$ value close to zero, incorrectly indicating no treatment effect.

Unless the assumption of proportional hazards is valid, the timing of an analysis can have a substantial effect on its conclusion, as we have demonstrated. The quotation of a single hazard ratio estimate from a survival study obviously presupposes at least approximate proportionality of hazards. Otherwise the trial findings have to be summarized through a series of hazard ratio estimates pertaining to different patient-time intervals or using some other non-constant presentation. Cox's regression analysis allows more complicated modelling, but it still relies on an assumption of proportional hazards and, when applied to two treatments in the absence of other covariates, it is essentially the same as the log-rank test.

For the trialist, the assumption of proportional hazards presents difficulties. Conventional calculations of sample size and study duration for survival trials are based on the proportional hazards assumption (Freedman, 1982; Machin and Campbell, 1987). Sequential designs tend also to require this assumption (Whitehead, 1993). The assumption of proportional hazards needs careful consideration at the design stage. Model checking of previous trials may cast doubts on its validity. Consideration of the mode of action of an experimental treatment might indicate, for example, that short-term mortality will be increased as the price for long-term benefit or, conversely, that benefits will affect short-term survival and quality of life but not the chances of survival beyond 1 year. In either case the assumption of proportional hazards is likely to be incorrect. An alternative summary measure of treatment advantage must be sought – not the (assumed constant) hazard ratio but perhaps the ratio of odds on survival past 1 year or the 'averaged' hazard ratio over 1 year. Calculations of sample size and study duration, and sequential methods can be based on these alternative measures. Either of the choices above will lead to studies requiring a substantial number of patients with at least 1 year of follow-up, or to sequential designs in which stopping is impossible before data on patients who have completed the whole of the 1st year of treatment begin to accumulate. In some diseases, it may be necessary to substitute 5 years for 1 year. Unless there are disastrous short-term results, these will have to be long studies, even if a sequential design is used.

In some cases, absolutely nothing will be known about the likely form of hazard ratios. The proportional hazards assumption perhaps leads to the default design. Post-trial model checking may either confirm this assumption or indicate that a further trial with a more suitable design is necessary. It is wise to plan for a long follow-up period if possible, especially if therapy is short term and irreversible (e.g. surgery) rather than long term with potential continuous harm (e.g. life-long drug therapy). This will allow any late evidence of treatment effect and of non-proportionality of hazards to emerge. We have shown how a mid-study review might be used so that the trial design might be revised to take account of emerging evidence about relative hazards. Such a review could be used as a prospective tool in a clinical trial – but caution is needed. First, at this relatively early stage, there will be little power to detect even serious departures from proportional hazards as being significant, and conversely misleadingly clear but non-significant patterns might arise. Second, as the review is not blind to treatment it could reveal such large survival differences that the trial has to be stopped. Rather than ignoring the latter possibility, it may be best to incorporate the review as the first (and possibly only) look within a formal sequential design. This will protect against inflation of risks of error and, in the case of pharmaceutical trials, satisfy regulatory requirements. We are not, on the other hand, going so far as to urge formal allowance for repetition of the model checking nor for its own effects on the final analysis.

If model checking confirms that the assumption of proportional hazards is at least approximately valid, then conventional analyses can be confidently presented; if serious departures from proportional hazards are present, then the situation is less clear. Few alternative methods have been extensively discussed in the statistical literature (Kalbfleisch and Prentice, 1981; Pepe and Fleming 1989).

The variability of data, especially early in a trial, and the fact that hazards may not be proportional means that clinicians must be cautious about accepting trial results at face value, especially when large early differences have led to early stopping of a study. Data monitoring committees in particular should be exceedingly cautious about stopping randomized trials when early effects occur, even if clearly significant by conventional tests. We can be more confident of a result when the trial size is very large and the follow-up time is long. Alas, this competes with other priorities in clinical research, such as the need to complete trials quickly and the need to ask the next, and most urgent, question.

### ACKNOWLEDGEMENTS

We are grateful to the EORTC Breast Cancer Co-operative Group for allowing us to use their trial (10792) as an illustrative example and especially to Professor Robert Rubens, Professor Harry Bartelink and Dr Richard Sylvester for releasing the original data on which our analyses are based.

### REFERENCES

Collett D (1994) *Modelling Survival Data in Medical Research.* Chapman & Hall: London

Cox DR (1972) Regression models and life-tables. *J R Statist Soc* **B34**: 187–202

Fayers PM, Cooke PA, Machin D, Donaldson N, Whitehead J, Ritchie A, Oliver RTD, Members of the Urological Working Party Renal Carcinoma Subgroup and Yuen P (1994) On the development of the Medical Research Council trial of α-Interferon in metastatic renal carcinoma. *Stats Med* **13**: 2249–2260

Freedman LS (1982) Tables of the number of patients required in clinical trials using the logrank test. *Stats Med* **1**: 121–129

Gould AL (1992) Interim analysis for monitoring clinical trials that do not materially affect the type I error rate. *Stats Med* **11**: 55–66

Kalbfleisch JD and Prentice RL (1981) Estimation of the average hazard ratio. *Biometrika* **68**: 105–112

Kaplan EL and Meier P (1958) Nonparametric estimation from incomplete observations. *Am Stat Assoc J* **53**: 457–481

Machin D and Campbell MJ (1987) *Statistical Tables for the Design of Clinical Trials.* Blackwell Scientific Publications: Oxford

Parmar MKB and Machin D (1995) *Survival Analysis: A Practical Approach.* Wiley: Chichester

© Cancer Research Campaign 1997

Pepe MS and Fleming TR (1989) Weighted Kaplan–Meier statistics: a class of distance tests for censored survival data. *Biometrics* **45**: 497–507

Peto R and Peto J (1972) Asymptotically efficient rank invariant test procedures (with discussion). *J R Stat Soc Series A* **135**: 185–206

Peto R, Pike MC, Armitage P, Breslow NE, Cox DR, Howard SV, Mantel N, McPherson K, Peto J and Smith PG (1977) Design and analysis of randomised clinical trials requiring prolonged observations of each patient. II. Analysis and examples. *Br J Cancer* **35**: 1–39

Rubens RD, Bartelink H, Engelsman E, Hayward JL, Rotmensz N, Sylvester R, Van Der Scheuren E, Papadiamantis J, Vasskaros SD, Wildiers J and Winter PJ (1989) Locally advanced breast cancer: the contribution of cytotoxic and endocrine treatment to radiotherapy. An EORTC Breast Cancer Co-operative Group Trial (10792). *Eur J Cancer Clin Oncol* **25**: 667–678

Souhami R (1994) The clinical importance of early stopping of randomised trials in cancer treatments. *Stats Med* **13**: 1293–1295

Sylvester R, Bartelink H and Rubens R (1994) A reversal of fortune: practical problems in the monitoring and interpretation of an EORTC breast cancer trial. *Stats Med* **13**: 1329–1335

Whitehead J (1993) Interim analyses and stopping rules in cancer clinical trials. *Br J Cancer* **68**: 1179–1185

© Cancer Research Campaign 1997